# Pyogenic Liver Abscess Secondary to Appendicitis

**DOI:** 10.7759/cureus.19188

**Published:** 2021-11-01

**Authors:** Brooke E Kania, Jalal Koj, Alisa Farokhian, Nader Mekheal, Angelo Bellardini

**Affiliations:** 1 Internal Medicine, St. Joseph's University Medical Center, Paterson, USA

**Keywords:** hepatic abscess, right upper quadrant, abdominal pain, acute appendicitis, pyogenic liver abscess

## Abstract

A pyogenic liver abscess secondary to appendicitis infection is a rare manifestation that has not been well illustrated in the United States due to its infrequency and the variability of each clinical presentation. Here, we discuss a 55-year-old male who presented with abdominal pain, fever, chills, and weight loss and was found to have a pyogenic liver abscess suspected secondary to radiographic-proven acute appendicitis. The purpose of this article is to describe a patient who presented with noteworthy clinical features and a rare cause of hepatic abscess, to aid in the treatment and diagnosis of future patients.

## Introduction

A pyogenic liver abscess represents a rare, yet dangerous manifestation that can be due to cryptogenic causes, pyemia through the portal vein, bowel leak, biliary infection, or penetrating trauma [1]. Patients who present with this diagnosis are typically males averaging 62 years old, and risk factors include prior liver transplant, malignancy, diabetes mellitus, or autoimmune disorders [1]. The clinical presentation of hepatic abscesses is varied depending on the source of infection; however, many of the presenting symptoms are vague in helping identify the source of infection. Clinical symptoms include fevers and chills and abdominal pain with associated nausea, vomiting, and weight loss [1-3]. As these patients may present with an ambiguous clinical picture, obtaining a thorough history is important. As the incidence of pyogenic liver abscesses is relatively low, the pathophysiology of this disease process has not been sufficiently researched [1]. A pyogenic liver abscess can manifest differently depending on the underlying source of infection. Mortality rates continue to decrease with better management of this disease, especially with proper antibiotics and abscess drainage [1-2].

## Case presentation

A 55-year-old Hispanic man with no significant past medical history presented to the emergency department (ED), with a 10-day history of worsening right upper quadrant and epigastric abdominal pain. The pain was described as intermittent, non-radiating, and “squeezing,” 10/10 in severity, worsening following meals, partially relieved with Pepcid, associated with nausea, unintentional weight loss, fevers, chills, and night sweats. The patient reported decreased appetite and weight loss of 10-11 pounds over 10 days. He returned from a trip to Costa Rica four months ago.

In the ED, vitals were notable for a blood pressure of 99/68 mmHg, which the patient reported is his baseline. The physical exam was significant for mild tenderness to palpation of the epigastric area, with negative rigidity, rebound, Murphy’s sign, or Rovsing’s sign. The patient was diaphoretic, and there was no evidence of scleral icterus. Laboratory studies were notable for leukocytosis with left-shift and bandemia with a white blood cell count of 17.1x103/mm^3^, absolute neutrophil count of 14.54, and 11% bands, total bilirubin was elevated at 1.5 mg/dL, and alkaline phosphatase was elevated at 290 unit/L. The patient had low albumin and total protein values, which were 3.1 g/dL and 5.8 g/dL, respectively.

**Figure 1:**
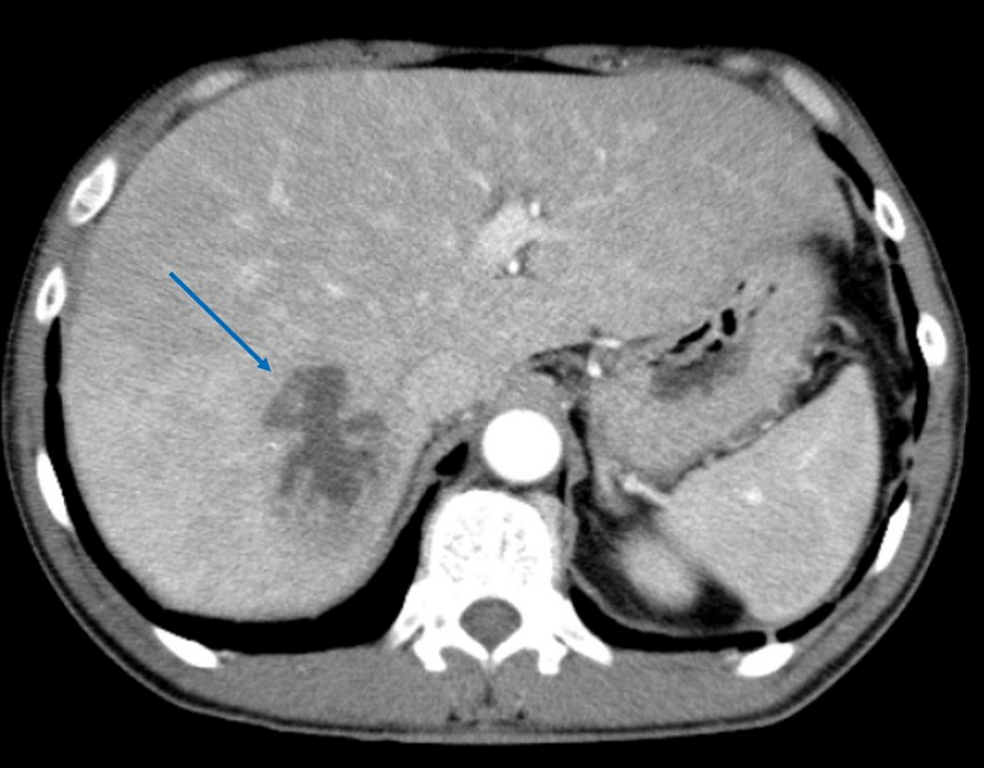
Computed tomography (CT) abdomen and pelvis with contrast The blue arrow indicates a septated hypodense mass in the right liver lobe, most likely representative of an abscess.

A right upper quadrant ultrasound (US) was performed, showing a liver of 16.8 cm and a common bile duct of 3.8 mm with no intrahepatic biliary dilatation and no evidence of ascites or gallbladder wall thickening or stones. Subsequently, a computed tomography (CT) scan of the abdomen and pelvis was significant for a septated hypodense mass measuring 6 cm in the right liver lobe, most likely representative of an abscess but not excluding hydatid disease or necrotic neoplasm (Figure 1). There was no comment on the appendix in the CT report. At this time, the patient was admitted to the hospital for further management of his pyogenic liver abscess.

**Figure 2:**
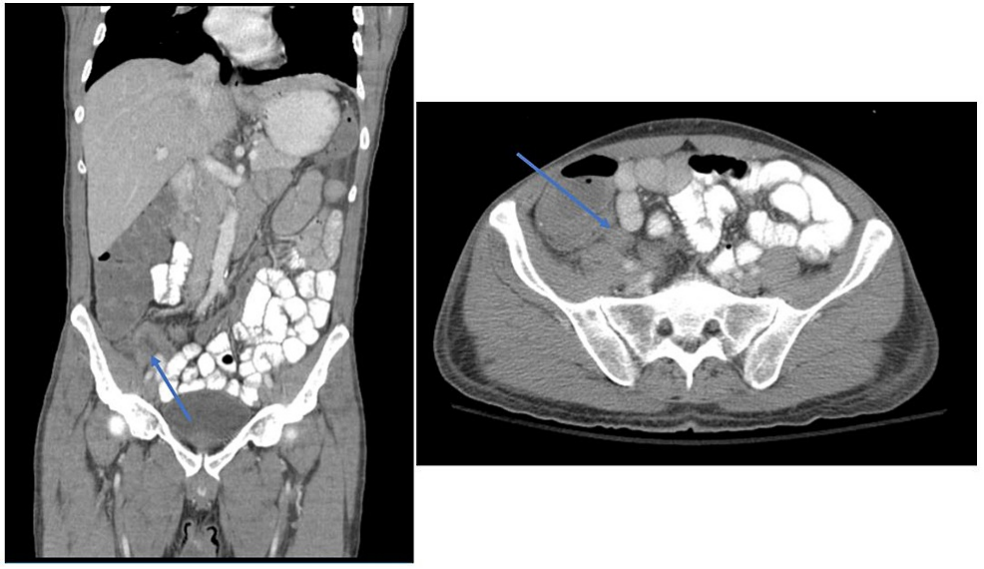
Repeat computed tomography (CT) abdomen and pelvis with contrast Blue arrows represent a mildly thickened appendix with trace inflammatory changes.

The admitting team transitioned the patient from intravenous Zosyn to empiric antibiotic coverage with intravenous ceftriaxone 2 g daily and intravenous metronidazole 500 mg every eight hours. Surgery delayed the drainage of the patient’s abscess until his Echinococcus lab result came back negative. During this waiting period, the patient had persistent leukocytosis, and repeat imaging was performed. Significant new findings of a mildly thickened appendix and trace inflammatory changes with acute appendicitis were not excluded (Figure 2). Seven days following admission, the patient proceeded with interventional radiology (IR)-guided drainage of his abscess, where 30 ccs of bloody purulent fluid were aspirated and sent for analysis. He was ultimately discharged home with his percutaneous drain and close outpatient follow-up.

## Discussion

A pyogenic abscess is a rare manifestation, with an incidence of approximately 3.6 per 100,000 population [1]. Patients who develop these abscesses have a mean age of 62.4 years old +/- 14 years, with males having predominance over females (3.3 vs 1.3 per 100,000) [1]. Risk factors for this disease process include liver transplantation history, history of malignancy, diabetes mellitus, history of alcoholism, as well as autoimmune processes such as rheumatoid arthritis and systemic lupus erythematosus [1]. At 55 years old, our patient was younger than the expected age. Our patient had a history of alcohol use with an average of one to two beers daily; however, since he did not follow regularly with a primary care physician, it is difficult to ascertain whether he had other underlying disease processes that could have contributed to his abscess formation.

Liver abscesses can develop from biliary infections, bowel leakage, or surgical penetration. In a retrospective study, more than half of the patients found to have a liver abscess had no primary cause for development, and 24% were found to have an abscess secondary to direct spread from biliary infection, including malignant obstructive process and gallstones [1]. In this same study, 20% had non-biliary sources, including bowel perforation, neutropenia, pneumonia, and/or empyema, perinephric abscess, endocarditis, dental abscess, traumatic liver injury, and appendicitis [1]. Of these patients, only 1% were found to have pyogenic abscess seeding from appendicitis. Our patient had an underlying acute appendicitis infection found multiple days following his admission via repeat imaging, thought to have seeded to the patient’s liver, causing the abscess. Especially considering the patient’s negative signs and symptoms for appendicitis, his cause for his abscess could have easily been missed without proper imaging.

Patients with a pyogenic liver abscess have been shown to present with fever (85%), chills (73.6%), abdominal pain (52.8%), fatigue (58.3%), weight loss (27.8%), nausea (31.9%), vomiting (20.8%), appetite loss (19.4%), jaundice (12.5%), and myalgias (16.7%) [2-3]. Our patient acknowledged fever, chills, abdominal pain, weight loss, nausea, and appetite loss when he presented to the ED. These symptoms are rather non-specific when it comes to pyogenic liver abscesses, as they are common with most abdominal infections. Signs that have been commonly found with pyogenic liver abscess patients include fever with a documented temperature greater or equal to 38.1 degrees Celsius (90%), abdominal tenderness to palpation (51.4%), hepatomegaly (15.3%), scleral icterus (13.9%), and altered mental status (12.5%) [2-3]. In comparison, our patient had documented fevers during his stay as well as abdominal tenderness to palpation.

Laboratory studies indicative of a liver abscess include hypoalbuminemia (70.2%-94%) as well as an elevated alkaline phosphatase level (67%-73%), both of which were exhibited in our patient [2,4]. As bacteremia can contribute to liver abscess development, blood cultures are important to obtain. In a retrospective study, bacterial cultures were found to be positive in 58% of patients with pyogenic liver abscesses, most commonly with Klebsiella species (13%) followed by Escherichia coli (20%) and then Bacteroides fragilis (13%) and Streptococcus milleri (8%); however, this study was conducted in China where there have been higher incidences of Klebsiella bacteremia-associated pyogenic abscesses compared to the United States [4]. No bacterial growth was observed upon obtaining the blood cultures of our patient, which provided evidence toward an alternative source of infection.

Given our patient’s travel history to Costa Rica, we could not exclude Entamoeba histolytica as a source of infection. Although his serology testing was ultimately negative and in the United States, amebic liver abscesses are rare due to less fecal-oral transmission, those who travel to Central America should be considered due to the higher prevalence in these areas [5].

As for imaging studies, both US and CT have identified liver abscesses and can be utilized as diagnostic methods [6]. Studies have shown that CT is more sensitive (sensitivity 97%) when compared to US studies (sensitivity 85.5%) [6-8]. In a retrospective analysis, 99% of patients with a pyogenic liver abscess had positive image findings on CT, and the 1% that was not identified was suspected to be due to non-enhanced imaging [1].

On ultrasound, micro-abscesses measuring less than 2 centimeters in size can appear as hypoechoic nodules or, alternatively, as ill-defined areas of hepatic echogenicity, and larger abscesses can vary from hypoechoic to hyperechoic masses [8]. When evaluating patients for a pyogenic liver abscess with CT imaging with contrast enhancement, abscesses can appear as small, well-defined hypoattenuating lesions, with faint rim enhancement and possible perilesional edema [8]. The majority of these abscesses will be found in the right liver lobe, with 77% of these lesions being solitary in nature [2]. Our patient had an absence of abnormal findings on US imaging and clear findings in the right liver lobe on CT, consistent with the increased sensitivity of the CT imaging modality.

Management is dependent on the source of infection. A meta-analysis identified that patients treated via percutaneous catheter drainage had superior outcomes in clinical improvement and days to achieve a 50% reduction in lesion size when compared to percutaneous needle aspiration [9]. Patients who completed percutaneous liver aspiration have been shown to have overall lower mortality rates [1]. Broad-spectrum antibiotics should be administered when suspecting a patient has a liver abscess, specifically with a penicillin-based antibiotic, aminoglycoside, and metronidazole (with appropriate modification towards culture results and patient response) [10]. Our patient clinically improved following antibiotics; however, once we obtained source control with abscess drainage, he had further improvement warranting his discharge from the hospital.

The overall prognosis of patients with pyogenic liver abscess has improved, where older studies have reported 11%-31% mortality and more recent studies show only 2.5%-5.6% mortality [1-2]. Both patients who were diagnosed at an older age (65-84 years old versus 18-34 years old) and those who developed bacteremia had an increased risk of mortality [1]. Our patient had rapid clinical improvement following his abscess drainage and with the use of empiric antibiotics, likely due to his younger age and absence of bacteremia.

## Conclusions

A pyogenic liver abscess represents a rare manifestation that can be caused by a variety of sources, including biliary, gastrointestinal perforation, through the portal circulation, injury to the liver, or seeding from alternative infections. A very small number of patients, including the patient presented above, has been shown to have a pyogenic liver abscess from suspected seeding from appendicitis. Common manifestations include subjective fever, chills, abdominal pain, leukocytosis, hypoalbuminemia, elevated alkaline phosphatase, and variable findings on imaging. Standard therapy shows improved outcomes with percutaneous drainage of the abscess and empiric broad-spectrum antibiotics until the underlying source of infection is identified. The goal of this case report is to complete a literature review of this rare clinical picture and improve diagnostic criteria for pyogenic liver abscesses by considering this as a possible complication of appendicitis. With additional knowledge of this disease process and further research, this may lead to an earlier diagnosis, possible prevention, and thus a continued reduction in incidence and mortality rates for these patients.
